# A Comparative Transcriptome Between Anti-drug Sensitive and Resistant *Candida auris* in China

**DOI:** 10.3389/fmicb.2021.708009

**Published:** 2021-07-16

**Authors:** Wenkai Zhou, Xiuzhen Li, Yiqing Lin, Wei Yan, Shuling Jiang, Xiaotian Huang, Xinglong Yang, Dan Qiao, Na Li

**Affiliations:** ^1^The First Affiliated Hospital of Nanchang University, Nanchang, China; ^2^Department of Medical Microbiology, School of Medicine, Nanchang University, Nanchang, China; ^3^Department of Laboratory Medicine, Ruijin Hospital, Shanghai Jiao Tong University School of Medicine, Shanghai, China

**Keywords:** *Candida auris*, RNA-seq, drug resistance, transcriptome, virulence

## Abstract

*Candida auris* emerged as a pathogenic species of fungus that causes severe and invasive outbreaks worldwide. The fungus exhibits high intrinsic resistance rates to various first-line antifungals, and the underlying molecular mechanism responsible for its multidrug resistance is still unclear. In this study, a transcriptomic analysis was performed between two *C. auris* isolates that exhibited different anti-drug patterns by RNA-sequencing, namely, CX1 (anti-drug sensitive) and CX2 (resistant). Transcriptomic analysis results revealed 541 upregulated and 453 downregulated genes in the resistant *C. auris* strain compared with the susceptible strain. In addition, our findings highlight the presence of potential differentially expressed genes (DEGs), which may play a role in drug resistance, including genes involved in ergosterol and efflux pump biosynthesis such as *SNQ2*, *CDR4*, *ARB1*, *MDR1*, *MRR1*, and *ERG* genes. We also found that Hsp related genes were upregulated for expression in the anti-drug-resistant strain. Biofilm formation and growth conditions were also compared between the two isolates. Our study provides novel clues for future studies in terms of understanding multidrug resistance mechanisms of *C. auris* strains.

## Introduction

*Candida auris* is a species of fungus that was firstly isolated from a patient’s external ear canal in Tokyo, Japan and represented a novel species within this genus in 2009 (Satoh et al., 2009). However, a study performed in 2011 revealed that *C. auris* already existed as a nosocomial infective agent in South Korea since 1996, in a case that the infection was treated as unidentified yeasts and which eventually caused nosocomial fungemia in a 1-year-old girl (Lee et al., 2011). Since its first description in 2009, the presence of *C. auris* was soon reported in all continents, except Antarctica, and which caused infections in more than 40 countries (Chow et al., 2018; Rhodes and Fisher, 2019; Vila et al., 2020). Genomic epidemiology revealed that *C. auris* exhibits five genetically diverse clades by genome sequencing. Consequently, this species was divided according to the following geographical clusters: Clade I (South Asian), Clade II (East Asian), Clade III (African), Clade IV (South American), and Clade V (Iran). Clade V was isolated from a patient in Iran and is the most recently identified clade (Lockhart et al., 2017; Chow et al., 2019). Genetic sequences of isolates from patients or environments are very important to trace the transmission of *C. auris* among different countries, hospitals, and perhaps different patients’ parts. What makes this species so important is its ability to spread and cause nosocomial outbreaks in hospitals and healthcare facilities in a similar manner to bacteria (Schelenz et al., 2016; Adams et al., 2018; Eyre et al., 2018; Ruiz-Gaitan et al., 2018, 2019; Armstrong et al., 2019; O’Connor et al., 2019). In fact, a recent review showed that the majority of the recovery sites in *C. auris* outbreaks involved candidemia (blood) and skin, which in turn underlines the ability of *C. auris* to induce invasive infections and colonization (Vila et al., 2020). Misidentification of *C. auris*, which unavoidably induces inappropriate and delayed treatment, is another important reason for these outbreaks. Unlike other yeasts, *C. auris* has often been confused with other pathogens such as *C. parapsilosis*, *C. haemulonii*, and *C. sake* by commercial systems (Lee et al., 2011; Kathuria et al., 2015; Mizusawa et al., 2017; Adams et al., 2018; Snayd et al., 2018; ElBaradei, 2020). Reliable identification methods involve systems with updated databases and molecular methods, which target the specific sequences of *C. auris* (Kathuria et al., 2015; Kordalewska et al., 2017; Sexton et al., 2018; Forsberg et al., 2019; Lima et al., 2019; Lone and Ahmad, 2019). However, the most worrying characteristic of *C. auris* pertains to its intrinsic or required resistance to a variety of first-line antifungals commonly used in clinical settings, including the three main categories of antifungals, i.e., azoles, echinocandins, and polyenes, thus limiting the underlying treatment options (Adams et al., 2018; Chaabane et al., 2019; Wickes, 2020). As a result, the misidentification and multidrug resistance of *C. auris* have both been prominent factors that enhance the ability of this fungus to cause infections with significant patient mortality, especially in patients that already suffer from concurrent diseases or receive invasive treatments (Lockhart et al., 2017; Armstrong et al., 2019; de Jong and Hagen, 2019; Taori et al., 2019).

Despite the fact that the multidrug resistance of *C. auris* has been a prevalent concern, there have only been a limited number of research studies performed to expound its mechanisms of antifungal resistance, and thus, these molecular mechanisms remain unknown (Chaabane et al., 2019; Kean and Ramage, 2019). For instance, several studies have focused on its phenotype. However, the majority of studies on antifungal resistance mechanisms are based on drug resistance-related genes in non-auris *Candida* species that have been previously reported (Arastehfar et al., 2020; Lee et al., 2020). Pertaining to azoles resistance, mutations in *ERG11* and *ERG11* overexpression play a distinct role in *C. auris* that facilitate drug target alteration and overexpression, respectively (Pristov and Ghannoum, 2019). Another common mechanism for azole resistance in *C. auris* involves efflux pumps enhanced overexpression including Major Facilitator Superfamily (MFS) and ATP Binding Cassette (ABC) that accelerate drug efflux (Kean et al., 2018; Lee et al., 2020). Echinocandin resistance in *C. auris* is the result of mutations in *FKS1* due to drug target alterations (Pristov and Ghannoum, 2019). However, the resistance mechanism to amphotericin B is still not confirmed, and it cannot be explained by any of the proposed mechanisms thus far. Apart from the pathways that the above genes participate in, certain genes play a role in triggering stress responses such as *HSP90*, which also confer resistance (Lee et al., 2020).

In this study, we performed transcriptome analysis on two *C. auris* strains using RNA-sequencing (RNA-seq). One strain showed elevated minimum inhibitory concentration (MIC) in fluconazole and micafungin, whereas the other strain was a susceptible strain. Until now, a limited number of research studies have been performed with RNA-seq between susceptible and resistant *C. auris* strains without imposing any conditions in China. Consequently, through the analysis of gene expression differences, we aimed to explore the resistance mechanism and identify genes that may play an important part of this process.

## Materials and Methods

### Bacterial Strains and Identification

The first *C. auris* strain used in this study was isolated from the environment in China and was named CX1. The other *C. auris* strain was acquired from the NCCLs (National Center for Clinical Laboratories), was isolated from a patient’s ascitic fluid, and was named CX2. Two isolates were identified as *C. auris* by sequencing ribosomal DNA internal transcribed spacer (ITS) combined with using matrix-assisted laser desorption/ionization time of flight mass spectrometry (Bruker Daltonics, Germany) (Schoch et al., 2012; Fraser et al., 2016). The Ethics Committee of The First Affiliated Hospital of Nanchang University (approval no. 2016026) approved the present study. All participants provided a written informed consent to participate in this study.

### Antifungal Susceptibility Testing

*In vitro* antifungal susceptibility testing was performed with YeastOne plate (Thermo Fisher, United States) on three replicates of two strains according to the Clinical and Laboratory Standards Institute (CLSI) broth microdilution method M27-A3 (Clinical and Laboratory Standards Institute [CLSI], 2008). Several fully isolated strains were also selected from a yeast isolate of 24 h pure yeast culture species, emulsified in sterile water, and mixed with vortex. Then, we adjusted yeast suspension to 0.5 McFarland and Pipetted 20 μl to 11 ml fungus inoculation broth. Broth suspension (100 μl) was pipetted onto the drug sensitive plate and incubated in 35°C for 24 h. Finally, SensititreVizion system (Thermo Fisher, United States) was used to read the test results of the drug sensitive plate. The activities of nine antifungals against two *C. auris* isolates were tested, including fluconazole, itraconazole, voriconazole, posaconazole, micafungin, anidulafungin, caspofungin, amphotericin B, and 5-flucytosine. The MIC endpoints were interpreted in tentative breakpoints proposed by CDC^1^, as follows: ≥32 for fluconazole, ≥2 for amphotericin B, ≥4 for anidulafungin and micafungin, and ≥2 for caspofungin.

### RNA Extraction

*Candida auris* cells were inoculated in yeast–peptone–dextrose (YPD) broth medium with constant shaking at 220 rpm at 30S°C for 18 h. The fungus was collected at an approximate OD 600 = 0.7, transferred to the EP tube, and resuspended in 50 μl of sterile water preheated at 30°C. Cells were cooled quickly in the liquid nitrogen and grinded to powder in the pre-cooled grinding tool. Then, we added 1 ml of Trizol solution (Invitrogen, United Kingdom), grinded, sealed the mortar with tin foil, and let it stand. When the Trizol–bacteria mixture became liquid, we gently grinded this mixture again. Ribozyme-free pipette tips were used to suck the Trizo-bacterial mixture into new ribozyme-free 1.5 ml EP tubes, which were subsequently centrifuged at 12,000 rpm for 10 min at 4°C. We pipetted the water phase to new 1.5 ml EP tubes and added an equal volume of 25:24:1 phenol/chloroform/isoamyl alcohol, which was vigorously shook for 10 s and centrifuged at 4°C, 12,000 rpm for 5 min. Water phase was transferred to new 1.5 ml EP tubes, and an equal volume of isopropanol was added into an ice-bath for 10 min. We then centrifuged the tubes at 12,000 rpm at 4°C for 5 min, and we discarded the supernatant. The total RNA was then washed with 75% ethanol and centrifuged at 12,000 rpm for 5 min, and the supernatant was discarded. Total RNA was dried for 5–10 min, resuspended in 25 μl DEPC water, and temporarily stored at −20°C. Following this protocol for obtaining the total RNA, we used electrophoresis to observe the integrity of the RNA using 1.0% agarose gel. Quality and concentration of the isolated RNA were assessed by NanoDrop 2000c (Thermo Scientific, United Kingdom). CX1 and CX2 isolates were cultured in triplicates named CX1-1, CX1-2, CX1-3 and CX2-1, CX2-2, CX2-3, respectively.

### Transcriptome Analysis

In total, six samples and three biological replicates for two strains were sent for cDNA library construction, transcriptome sequencing, and analysis conducted by OE biotech Co., Ltd. (Shanghai, China). Furthermore, TruSeq Stranded mRNA LT Sample Prep Kit (Illumina, San Diego, CA, United States) was used to construct cDNA library in accordance with the manufacturer’s instructions. After the constructed library was qualified with Agilent 2100 Bioanalyzer, it was sequenced using Illumina HiSeq X Tento to generate 150 bp paired-end reads. Raw reads of fast format were preprocessed using Trimmomatic (Bolger et al., 2014), and the number of reads in the whole quality control was statistically summarized. Each sample’s clean reads remained after quality pretreatment steps, including the removal of reads containing adaptor, low quality reads, and bases arranged in different ways from the 3′ end and 5′ end. HISAT2 (Kim et al., 2015) was used to compare the clean reads with the specific reference genome^2^ to obtain the position information on the reference genome and the unique sequence feature information of the sequenced samples. The STRING (Search Tool for the Retrieval of Interacting Genes) database (Szklarczyk et al., 2019) was used to construct a protein–protein association network and to visualize the interactome network.

### DEG Analysis

Each gene’s FPKM (Roberts et al., 2011) value was calculated using Cufflinks (Trapnell et al., 2010), and its read counts were obtained by HTSeq-count (Anders et al., 2015). Cluster analysis was performed with the “pheatmap” package to explore gene expression pattern. Correlation test between samples was carried out using R language to calculate the Pearson correlation coefficient. DEseq (2012) R package was used to perform differential expression analysis. The threshold of significantly differential expression between samples was a false discovery rate (FDR) < 0.05 and fold change > 1.5.

### Functional Annotation

Gene Ontology (GO) enrichment and Kyoto Encyclopedia of Gene and Genomes (KEGG) (Kanehisa et al., 2008) pathway enrichment analysis of differentially expressed genes (DEGs) were performed using R based on the hypergeometric distribution. Biological process, cellular component, and molecular function enrichment analysis in GO level 2 were performed on DEGs using the fisher algorithm.

### Virulence Factor Prediction

We initially searched for genes associated with phospholipase, proteinase, hemolysin, adhesin, and biofilm formation in our transcriptome results. Subsequently, we searched for phospholipase, proteinase, hemolysin, and adhesin in Candida genome database (CGD). After retrieving related genes in other *Candida* species, we then searched for orthologous genes or best hits in *Candida auris*, shown in Supplementary Table 2. For biofilm formation, we downloaded the biofilm formation phenotype from CGD and performed a blastp with DEGs in the transcriptome. The criteria for filtering the blastp results were over 50% coverage and identity.

### Biofilm Formation Experiment and Growth Experiment

Prior to developing the biofilm, we treated the 96-well plate (Corning, United Kingdom) with fetal bovine serum, blocked at 4°C for 72 h, and then washed with sterile phosphate buffer saline. Candida was inoculated in YPD medium broth, incubated with shaking at 220 rpm at 30°C overnight, and then resuspended with Spider medium to make OD_600_ = 0.5. We added 200 μl bacterial solutions to each well in the experimental group and 200 μl Spider medium in the control group. The plate was incubated at 37°C under shaking at 200 rpm for 90 min and then washed with PBS. Each hole was added with 200 μl Spider medium and sealed with sealing film. After being incubated at 37°C under shaking at 200 rpm for 48 h, the culture medium was discarded, and each hole was washed with PBS. We fixed each well with 200 μl methanol for 30 min and then used 200 μl 11% crystal violet to stain after discarding fixative. Then, we absorbed the crystal violet and washed each well with PBS after washing with slow water flow and then decolorized each well with glacial acetic acid for 30 min. Finally, biofilm formation was measured according to spectrophotometric methods using microplate reader (Biotek, United States).

A spot dilution assay was performed to compare the growth status of the two *C. auris* isolates and *C. albicans*. Candida was incubated in YPD liquid medium with constant shaking at 220 rpm at 30°C overnight. Then, we collected the bacteria by centrifugation at 3,000 rpm, washed with sterile PBS, and resuspended with PBS. The yeast suspension was adjusted to an optical density (OD_600_) of 0.1 and was diluted by 10-fold serial in sterile PBS to a final OD_600_ of 10^–2^, 10^–3^, 10^–4^, 10^–5^, 10^–6^, and 10^–7^. A total of 1 μl suspension of each dilution was spotted on the YPD agar plate, and the plate was cultured at 30°C for 4 days before observing the growth differences of the two strains’ colonies.

## Results

### Antifungal Susceptibility

As demonstrated in Table 1, CX2 exhibits a higher MIC toward antifungals than CX1, except 5-flucytosine, itraconazole, and posaconazole, thus indicating that CX2 was a resistant strain as opposed to CX1. Apart from fluconazole (MIC = 2 μg/ml), CX1 exhibited high susceptibility to all three main categories of antifungals and 5-flucytosine. With respect to the antifungal susceptibility testing of azoles, fluconazole was the least active azole (MIC = 16 μg/ml) against CX2. Interestingly, CX2 exhibited higher susceptibility to itraconazole (MIC≤0.015 μg/ml) and posaconazole (≤0.008 μg/ml) compared to CX1. MICs of echinocandins were greater for CX2 compared to CX1: caspofungin (MIC = 1 μg/ml), anidulafungin (MIC = 2 μg/ml), and micafungin (MIC = 8 μg/ml). Furthermore, CX2 displayed significant resistance to micafungin, which may represent the underlying drug resistance mechanism. The MIC of amphotericin B was 1 μg/ml in CX2, whereas the MIC of 5-flucytosine was ≤0.06 μg/ml, both in CX1 and CX2 (Table 1).

**TABLE 1:** Antifungal susceptibility.

Strain	MIC, μ g/ml								
	FLZ	ITZ	VRZ	PSZ	MCF	ANF	CSF	AMB	FC
CX1	2	0.03	0.015	0.015	0.06	0.12	0.06	0.25	≤0.06
CX2	64	≤0.015	0.06	≤0.008	8	2	1	1	≤0.06

*FLZ, fluconazole; ITZ, itraconazole; VRZ, voriconazole; PSZ, posaconazole; MCF, micafungin; ANF, anidulafungin; CSF, caspofungin; AMB, amphotericin B; FC, 5-flucytosine.*

### Biofilm Formation and Growth Condition

As opposed to the two *C. auris* isolates that did not form any biofilm and were thus the same as the blank control, *C. albicans* (SC5314) formed biofilm in the 96-well plate (Supplementary Figure 1A). The spot assay was used to assess the growth status of the two *C. auris* isolates and *C. albicans* (SC5314) (Supplementary Figure 1B). No growth differences were found between the two *C. auris* strains and between *C. auris* and *C. albicans* strains.

### Transcriptome Analysis

In this study, we performed a comparative RNA-seq analysis on two clinical types (CX1 and CX2) including six samples (CX1-1, CX1-2, CX1-3, CX2-1, CX2-2, and CX2-3) to identify DEGs that may potentially facilitate drug-related resistance. The Illumina sequencing of the transcriptome of these six samples produced raw RNA-seq reads. To minimize the effect of data error in our results, we used the Trimmomatic software to pre-process the original data, and the number of reads in the whole quality control process was statistically summarized (Supplementary Table 1).

Hisat2 was used to sequence the aligned Clean Reads with the specified reference genome to obtain the location information on the reference genome or gene, as well as the sequence characteristic information that was unique to the sequencing sample (Supplementary Table 2). The percentage of raw reads of samples mapped to the *C. auris* genome was high (over 98% in total mapped reads), thus indicating that these samples were consistent with *C. auris*.

**FIGURE 1:**
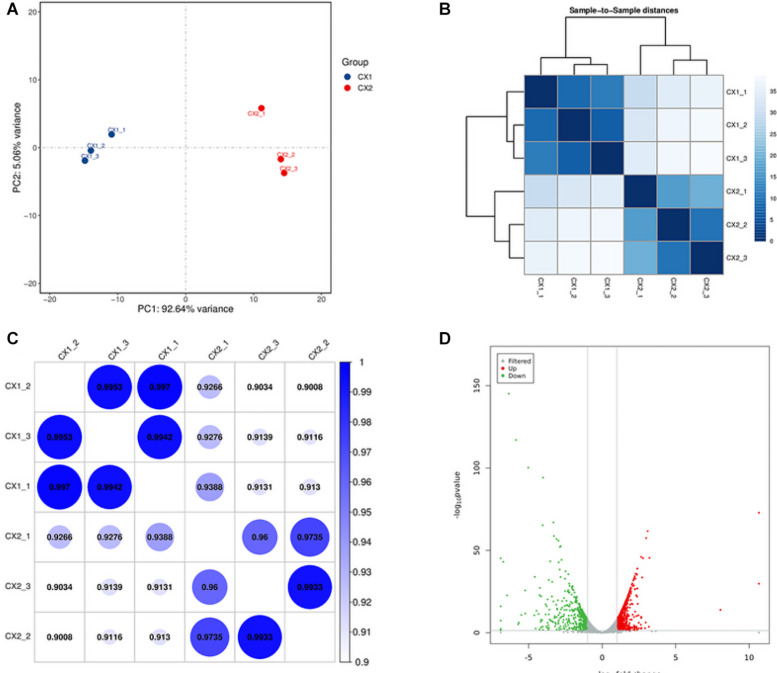
Validation of *C. auris* transcriptome. **(A)** Principal component analysis (PCA) plot showing the level of correlation among CX1 (blue) and CX2 (red). The closer the PCA plot distance is, the more similar the samples are. **(B)** Cluster analysis of gene expression data. The chromaticity of the color represents the size of the correlation coefficient. **(C)** Heat map of correlation coefficient between samples. The color represents the size of the correlation coefficient. **(D)** Volcano plot. Every dot represents a differentially expressed gene. Gray dots were non-significantly different genes, and the green and red dots were significantly upregulated different genes in CX1 and CX2, respectively.

Principal-component analysis and cluster analysis were used to demonstrate a visual representation of the correlation in transcriptome between the different replicates (Figures 1A,B). Samples from different isolates were clustered separately, whereas samples from the same isolates were clustered together. Furthermore, the protein coding gene expression level was used to generate a heatmap of correlation coefficient between samples (Figure 1C). The results obtained reflected a high-level of relevance among samples from the same strain and slight differences between CX1 and CX2.

**FIGURE 2:**
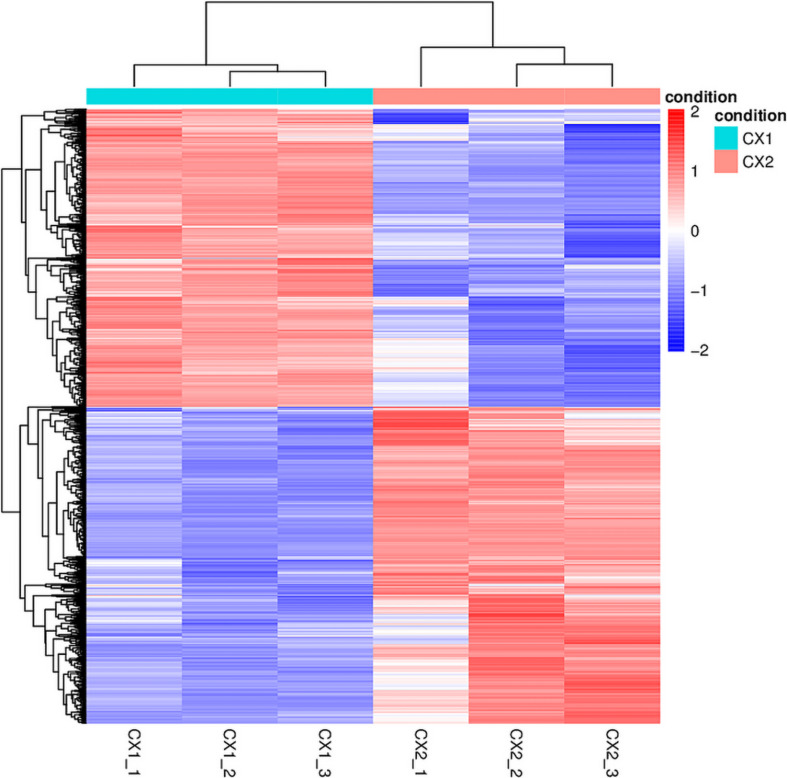
The heatmap of DEGs identified in this study. In the heatmap, rows in red and blue represent upregulated and downregulated genes, respectively. The different depth of colors represents different level of DEG expression.

RNA-seq data analysis clearly displayed a wide range of differences between CX1 and CX2 gene expression in the transcriptome. More specifically, genes with a 1.5-fold change (up or down) in the level of expression were considered to reflect differences in gene expression, and they were thus regarded as significantly regulated genes. A negative binomial distribution test revealed a *P*-value < 0.05. Nine hundred ninety-four statistically significant variation genes were found in expression between our samples, of which 541 were upregulated and 453 were downregulated (Supplementary Table 3). The volcano plot was used to demonstrate the number of significantly DEGs between CX1 and CX2 (Figure 1D). Clustering analysis of the differential expression patterns showed that the DEGs were consistent across replicates, with a significant variation between CX1 and CX2 (Figure 2).

### Enrichment Analysis of DEGs

**FIGURE 3:**
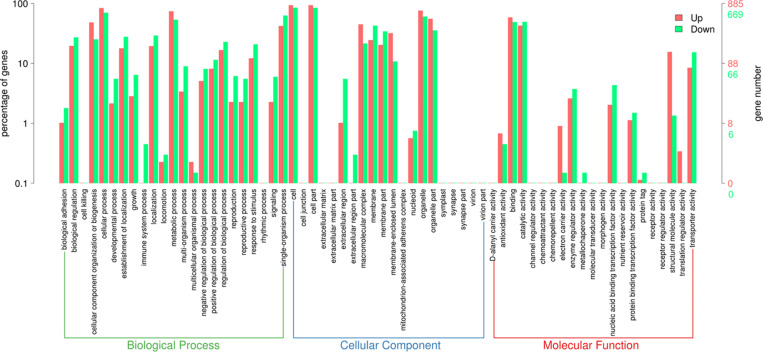
Gene Ontology (GO) term enrichment of differentially expressed genes between sensitive and resistant strains. Three GO categories including molecular function, cellular component, and biological process were used to classify the DEGs. Red represented upregulated genes, and green represented downregulated genes.

After obtaining the statistically DEGs, the GO enrichment analysis was carried out on the DEGs to describe their respective functions (combined with the GO annotation results). The DEGs could be divided into three main GO categories, i.e., biological process, cellular component, and molecular function (Figure 3). The 669 downregulated differential expression genes were assigned to 43 GO terms, including 21 biological processes, 11 cellular components, and 11 molecular functions. More specifically, these genes were distributed as follows: biological processes included cellular processes (70.3%), single-organism processes (62.9%), and metabolic processes (53.7%); cellular components associated with cells (85.2%), cell parts (84.6%), and organelles (60.5%); and molecular functions containing catalytic activity (49.3%) and binding (49.0%). In contrast, the 885 upregulated differential expression genes were assigned to 41 GO terms, including 20 biological processes, 10 cellular components, and 11 molecular functions. More specifically, these genes were distributed as follows: biological processes, including cellular processes (84.0%) and metabolic processes (74.4%); cellular components associated with cells (93.6%), cell parts (93.6%), and organelles (76.0%); molecular functions contributing to binding (58.8%) and catalytic activity (42.7%).

**FIGURE 4:**
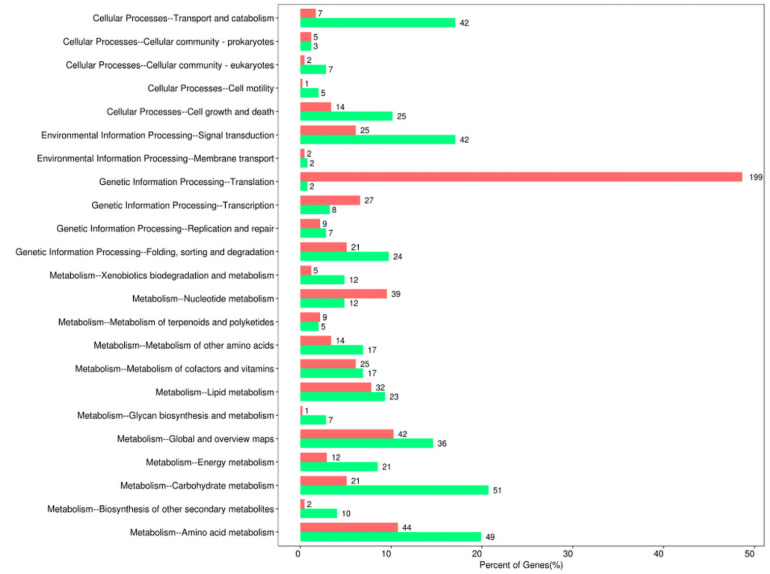
Kyoto Encyclopedia of Gene and Genomes (KEGG) pathway enrichment of differentially expressed genes between sensitive and resistant strains. Red represented upregulated genes, and green represented downregulated genes.

Moreover, the pathway-analysis of differentially expressed protein coding genes using the KEGG database (combined with KEGG annotation results) could reveal the relationship between drug resistance and cellular pathway changes. A total of 427 downregulated genes and 558 upregulated genes were categorized into known KEGG pathways. Among the 427 downregulated genes, 82 DEGs were distributed in cellular processes that were mainly sub-categorized under transport and catabolism (17.1%), and cell growth and death (10.2%). In addition, 44 DEGs were distributed in environmental information processing that were mainly sub-categorized under signal transduction (17.1%), whereas 41 DEGs were distributed in genetic information processing that were mainly sub-categorized under folding, sorting, and degradation (9.8%). Finally, 260 DEGs were distributed in metabolism, and they were mainly sub-categorized under carbohydrate metabolism (20.7%), amino acid metabolism (19.9%), and global and overview maps (14.6%). In contrast, among the 558 upregulated genes, 29 DEGs were distributed in cellular processes that were mainly sub-categorized under cell growth and death (3.4%), and 27 DEGs were distributed in environmental information processing that were mainly sub-categorized under signal transduction (6.1%). Furthermore, 256 DEGs were distributed in genetic information processing that were especially sub-categorized under translation (48.7%), and 246 DEGs were distributed in metabolism that were mainly sub-categorized under nucleotide metabolism (9.5%), amino acid metabolism (10.8%), and global and overview maps (10.3%) (Figure 4).

### Virulence Factors

A previous study performed showed that *C. auris* exhibit phospholipase, proteinase, and hemolysin activity *in vitro* (Kumar et al., 2015). In another study, *C. auris* were found to produce phospholipase and proteinase that varied by strain (Larkin et al., 2017). The draft genome of *C. auris* revealed that virulence may be caused as a result of its diverse transporters, secreted aspartyl proteinases, secreted lipases, phosphatases, phospholipase, mannosyl transferases, integrins, adhesins, and transcription factors (Chatterjee et al., 2015). In this study, we investigated phospholipase, proteinase, hemolysin, adhesin, and biofilm formation-related genes in CGD. Initially, we did not identify hemolysin-related genes in DEGs between sensitive and resistant strains. However, a certain number of DEGs exist, which were related with phospholipase, proteinase, adhesion, and biofilm formation (Table 2). In fact, Table 2 shows that a greater number of downregulated than upregulated genes were related with phospholipase, proteinase, and adhesin. These findings are consistent with the common knowledge that drug resistance strains possess fewer virulence factors and thus demonstrate wear virulence. Meanwhile, our findings regarding DEGs related with biofilm formation were inconsistent with previous results that suggested a slightly greater number of upregulated genes compared to downregulated genes.

**TABLE 2:** Upregulated and downregulated phospholipase-, proteinase-, adhesin-, and biofilm-associated genes (CX2 vs. CX1).

Gene-identifier	Gene symbol	Description	Function	Fold change (log_2_)
B9J08_004010	PLB1	Lysophospholipase 1	Phospholipase	–2.88
B9J08_003621	PLB3	Lysophospholipase 3	Phospholipase	–0.86
B9J08_003289	SPAC6G10.03c	Probable cardiolipin-specific deacylase, mitochondrial	Phospholipase	–0.59
B9J08_003446	RAS1	Ras-like protein 1	Phospholipase	–0.69
B9J08_005379	ATG15	Putative lipase ATG15	Phospholipase	–0.92
B9J08_003305	CDC25	Cell division control protein 25	Phospholipase	–0.98
B9J08_000458	CEK1	Extracellular signal-regulated kinase 1	Phospholipase	–0.86
B9J08_005379	ATG15	Putative lipase ATG15	Phospholipase	–0.92
B9J08_003361	RHO1	GTP-binding protein RHO1	Phospholipase	0.60
B9J08_003959	SLC1	Probable 1-acyl-sn-glycerol-3-phosphate acyltransferase	Phospholipase	0.69
B9J08_004606	YOR059C	Putative lipase YOR059C	Phospholipase	0.69
B9J08_001064	PGC1	Phosphatidylglycerol phospholipase C	Phospholipase	0.63
B9J08_003873	PLC1	1-Phosphatidylinositol 4,5-bisphosphate phosphodiesterase 1	Phospholipase	0.85
B9J08_000871	LAP3	Cysteine proteinase 1, mitochondrial	Proteinase	–1.25
B9J08_005051	YIL108W	Putative zinc metalloproteinase YIL108W	Proteinase	–0.73
B9J08_002962	LAP3	Cysteine proteinase 1, mitochondrial	Proteinase	–1.11
B9J08_003912	YPS1	Aspartic proteinase 3	Proteinase	–0.75
B9J08_001019	RRT12	Subtilase-type proteinase RRT12	Proteinase	1.27
B9J08_002266	TRY4	Transcriptional regulator of yeast form adherence 4	Adhesin	–1.17
B9J08_000829	TRY5	Transcriptional regulator of yeast form adherence 5	Adhesin	–2.76
B9J08_002582	ALS4	Agglutinin-like protein 4 (fragments)	Adhesin	8.05
B9J08_001242	PGA1	Predicted GPI-anchored protein 1	Adhesin	–0.61
B9J08_001958	SAP9	Candidapepsin-9	Adhesin	–1.08
B9J08_002075	SDS3	Transcriptional regulatory protein SDS3	Adhesin	–0.98
B9J08_002266	TRY4	Transcriptional regulator of yeast form adherence 4	Adhesin	–1.17
B9J08_000829	TRY5	Transcriptional regulator of yeast form adherence 5	Adhesin	–2.76
B9J08_001192	SNF2	Transcription regulatory protein SNF2	Adhesin	–0.86
B9J08_002529	BRG1	Biofilm regulator 1	Adhesin	–1.73
B9J08_002596	MP65	Cell surface mannoprotein MP65	Adhesin	–2.63
B9J08_003278	MCM1	Transcription factor of morphogenesis MCM1	Adhesin	–0.77
B9J08_003305	CDC25	Cell division control protein 25	Adhesin	–0.98
B9J08_003836	HXK1	*N*-Acetylglucosamine kinase 1	Adhesin	–0.73
B9J08_003920	PKH2	Serine/threonine-protein kinase PKH2	Adhesin	–0.76
B9J08_004027	WOR1	White-opaque regulator 1	Adhesin	–2.03
B9J08_000458	CEK1	Extracellular signal-regulated kinase 1	Adhesin	–0.86
B9J08_000675	FLO9	Flocculation protein FLO9	Adhesin	–3.28
B9J08_000447	CRZ2	Transcriptional regulator CRZ2	Adhesin	1.48
B9J08_000592	UME6	Transcriptional regulatory protein UME6	Adhesin	2.49
B9J08_001918	AHR1	Adhesion and hyphal regulator 1	Adhesin	2.38
B9J08_001940	HSP12	12 kDa heat shock protein	Adhesin	1.56
B9J08_002582	ALS4	Agglutinin-like protein 4 (fragments)	Adhesin	8.05
B9J08_003361	RHO1	GTP-binding protein RHO1	Adhesin	0.60
B9J08_003550	YWP1	Yeast-form wall protein 1	Adhesin	2.96
B9J08_003772	CZF1	Zinc cluster transcription factor CZF1	Adhesin	1.42
B9J08_005078	AAH1	Adenine deaminase	Adhesin	0.79
B9J08_005458	ASC1	Guanine nucleotide-binding protein subunit beta-like protein	Adhesin	1.58
B9J08_001196	CSA1	Cell wall protein 1	Biofilm formation	–1.00
B9J08_000458	CEK1	Extracellular signal-regulated kinase 1	Biofilm formation	–0.86
B9J08_002788	TPK2	cAMP-dependent protein kinase type 2	Biofilm formation	–1.29
B9J08_004015	GAM1	Glucoamylase 1	Biofilm formation	–0.73
B9J08_000003	zrt1	Zinc-regulated transporter 1	Biofilm formation	–1.41
B9J08_002763	FAA1	Long-chain-fatty-acid–CoA ligase 1	Biofilm formation	–0.70
B9J08_000860	TAF14	Transcription initiation factor TFIID subunit 14	Biofilm formation	–0.74
B9J08_000928	AQY1	Aquaporin-1	Biofilm formation	–1.90
B9J08_001383	ams1	Alpha-mannosidase	Biofilm formation	–1.86
B9J08_003563	ADH2	Alcohol dehydrogenase 2	Biofilm formation	–3.10
B9J08_001633	YMR315W	Uncharacterized protein YMR315W	Biofilm formation	–0.59
B9J08_004068	VPS4	Vacuolar protein sorting-associated protein 4	Biofilm formation	–1.06
B9J08_004334	PHO2	Regulatory protein PHO2	Biofilm formation	–1.09
B9J08_004477		Glucan 1,3-beta-glucosidase	Biofilm formation	–3.62
B9J08_005380	SUR7	Protein SUR7	Biofilm formation	–0.60
B9J08_003614	ADH2	Alcohol dehydrogenase 2	Biofilm formation	–3.61
B9J08_000384	EPD1	Protein EPD1	Biofilm formation	1.40
B9J08_000822	CBK1	Serine/threonine-protein kinase CBK1	Biofilm formation	0.60
B9J08_001624	ILS1	Isoleucine–tRNA ligase, cytoplasmic	Biofilm formation	1.28
B9J08_001686	RPS4A	40S ribosomal protein S4-A	Biofilm formation	1.39
B9J08_001939	RIX7	Ribosome biogenesis ATPase RIX7	Biofilm formation	1.18
B9J08_002042	At2g30170	Probable protein phosphatase 2C 26	Biofilm formation	1.44
B9J08_002043	ARO1	Pentafunctional AROM polypeptide	Biofilm formation	0.78
B9J08_002365	STH1	Nuclear protein STH1/NPS1	Biofilm formation	0.61
B9J08_002420	FAS2	Fatty acid synthase subunit alpha	Biofilm formation	0.90
B9J08_002855	PMA1	Plasma membrane ATPase 1	Biofilm formation	0.98
B9J08_003041	PDX1	Pyruvate dehydrogenase complex protein X component, mitochondrial	Biofilm formation	1.33
B9J08_003159	CPH1	Transcription factor CPH1	Biofilm formation	1.17
B9J08_003402	ZPR1	Zinc finger protein ZPR1	Biofilm formation	1.73
B9J08_003582	RPS4A	40S ribosomal protein S4-A	Biofilm formation	1.81
B9J08_003641	DUS3	tRNA-dihydrouridine(47) synthase [NAD(P)(+)]	Biofilm formation	1.12
B9J08_003772	CZF1	Zinc cluster transcription factor CZF1	Biofilm formation	1.42
B9J08_004640	SIM1	Secreted beta-glucosidase SIM1	Biofilm formation	0.79
B9J08_004918	HSP90	Heat shock protein 90 homolog	Biofilm formation	0.80
B9J08_005403	QDR3	MFS antiporter QDR3	Biofilm formation	0.84

### Protein Interaction Network

**FIGURE 5:**
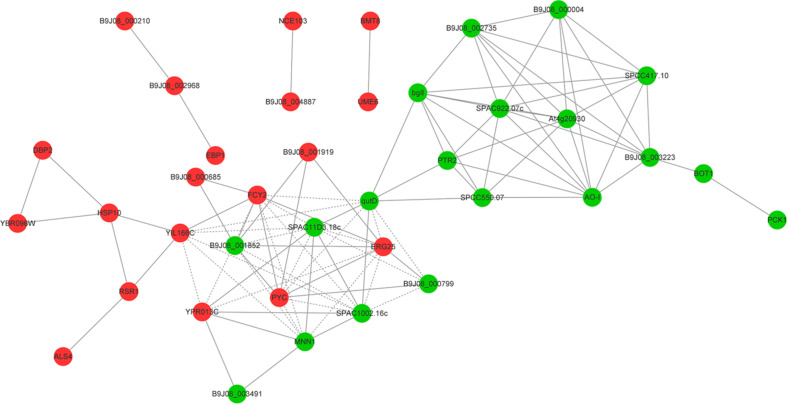
A predicted protein–protein interaction network. The top 20 upregulated DEGs and top 20 downregulated DEGs constitute the predicted protein interaction network using the STRING (Search Tool for the Retrieval of interacting Genes) database. The red and green nodes with gene names represented upregulated and downregulated differentially expressed genes, respectively.

In order to acquire a more accurate visualization of the molecular mechanism of multidrug resistance involved in *C. auris*, we used the STRING (Search Tool for the Retrieval of interacting Genes) database to generate a predicted protein interaction network that contained the top 20 downregulated DEGs and top 20 upregulated DEGs (Figure 5). The red and green nodes with gene names represented the upregulated and downregulated DEGs, respectively. The obtained picture shows that the proteins were divided into two clusters. Consequently, the core genes that more predicted associations with other genes and other remaining genes within the network were then investigated.

## Discussion

*Candida auris* is a pathogen that has been known for more than 10 years, yet it continuously causes outbreaks and exhibits alarmingly high drug resistance rates and even pan-drug resistance, which has not been efficiently studied (Wickes, 2020). Despite the high mortality rates caused by *Candida auris* infections, due to the rapid spread and frequent worldwide outbreaks caused by this fungus, only a small number of drugs can appropriately treat fungal infections. To understand the mechanism that facilitates drug resistance, we performed transcriptome of two *Candida auris* isolates, one of which was found to be resistant to fluconazole (MIC = 64) and micafungin (MIC = 8), and the other was found to be susceptible to antifungal drugs based on CDC reports (see text footnote 1). Therefore, we focused on the underlying mechanism of resistance to azoles and echinocandins in *Candida auris*. Due to the fact that the two *Candida auris* strains were unable to form biofilms, we searched the agglutinin-like sequence (*ALS*) genes in DEGs that play an important role in biofilm formation after attachment to abiotic surfaces (Hoyer and Cota, 2016). As expected, we only found *ALS4*, which is the most frequently expressed gene in the *ALS* gene family and which is differentially expressed between these two strains (but with low expression levels) (Monroy-Perez et al., 2012). The average value of *ALS4* gene expression in three biological replicates of CX1 was 0.012, whereas the average value of *ALS4* gene expression in three biological replicates of CX2 was 4.069. The significantly low expression level of *ALS* genes may be the reason for their inability to form biofilms. Enrichment analysis of DEGs revealed abundant differential pathways and gene functions between strains. The comparison of the GO classification enriched by upregulated and downregulated genes revealed that more upregulated genes were found than downregulated genes involved in electron carrier activity, structure molecule activity, and translation regulator activity, which may be related with transmembrane transporters such as efflux pumps. In addition, more downregulated genes were involved in immune system processes, extra cellular regions, extracellular region parts, and metallochaperone activity; however, further research is required to validate these findings. Nonetheless, pertaining to the KEGG pathway classification, more upregulated genes were found in resistant *Candida auris* with respect to translation, transduction, and nucleotide metabolism. However, more downregulated genes were involved in transport and catabolism, signal transduction, folding, sorting and degradation, and most metabolism pathways, a finding indicating that the metabolism of resistant strain is less active compared to susceptible strains or that a lower number of genes can in fact play a more significant role.

The azoles inhibit the activity of the lanosterol 14-α-demethylase encoded by *ERG11*, thus blocking ergosterol synthesis. Known mechanisms of resistance to azole include point mutations in *ERG11* that lead to a decrease in the affinity of drugs and enzymes. Furthermore, overexpression of *ERG11* and efflux pump genes can also cause a decrease in azole susceptibility (Lee et al., 2020). In our results, we investigated genes that are involved in steroid biosynthesis and efflux pumps pathways, which may in turn facilitate resistance to fluconazole. We identified genes that are involved in ergosterol and efflux pump biosynthesis that were upregulated expressed in resistant *Candida auris* strains including *SNQ2*, *CDR4*, *ARB1*, *MDR1*, *MRR1*, and 9 (*ERG1*, *ERG7*, *ERG11*, *ERG24*, *ERG25*, *ERG6*, *ERG2*, *ERG3*, and *ERG5*) of 13 of the ergosterol synthesis genes, which were consistent with previous studies performed (Chowdhary et al., 2018; Kean et al., 2018; Rybak et al., 2019). SNQ2, CDR4, MDR1, and ARB1 were found to be present and upregulated in drug-resistant isolates of *C. auris* (Munoz et al., 2018; Wasi et al., 2019). The ATP binding cassette (ABC) superfamily and major facilitator (MF) superfamily are the two main classes of efflux pumps. RNA-seq analysis indicated that the downregulated efflux pump genes such as an ABC transporter gene (*ABCF3*) and two MFS transporter genes (*hxnP* and *ecdD*) may play an atypical role. However, these genes have not been previously reported, and thus, further investigating their properties is imperative. In addition to *ERG11* and efflux pump overexpression, we also identified point mutations in ERG11. The amino acid substitutions at Y132F, K143R, and F126L were considered the cause of the azole resistance in *C. auris*, with Y132F being the most widespread mutation associated with azole resistance (Lee et al., 2021). We found point mutations at T395A which led to the amino acid substitution at Y132F in all three biological replicates of drug-resistant strains; this was surprisingly consistent with previous studies. However, we did not identify any point mutations in ERG11 in the susceptible strains.

Echinocandins exert their antifungal effect by inhibiting beta (1,3) D-glucan synthase encoded by *FKS*, thus causing a defective cell wall. Unlike azole resistance, several studies exist, which demonstrate that amino acid substitutions at S639F, S639P, and S639Y in *FKS1* simply lead to elevated MIC levels of echinocandins, as opposed to the role played by efflux pumps (Berkow and Lockhart, 2018; Chowdhary et al., 2018; Kordalewska et al., 2018; Lee et al., 2021). However, our study detected one amino acid substitution at R464P in the sensitive strain, in addition to three point mutations, T588C, A897G, and G2458T (same-sense mutations), in the resistant strain. We tried to explain the high MIC of micafungin (MIC = 8 μg/ml) in CX2 with HSP-associated genes. Heat shock proteins (HSPs) contain a large number of proteins that are distributed widely and are involved in many cellular pathways to modulate stress responses (Gong et al., 2017; Lee et al., 2020). In transcriptome analysis, we found that all HSP related genes were upregulated in resistant *Candida auris* strains such as *HSP90*, a strain that has been studied before. However, the exact role of HSPs in the resistance mechanism needs to be further studied.

In addition, this study assessed the potential role of protein modification genes in the underlying resistance mechanism. Epigenetics has been continuously studied in mammals; however, few studies have been performed that focus on fungal resistance mechanisms and epigenetics (Madhani, 2021). Therefore, we searched for genes that are involved in methylation, SUMOylation, ubiquitination, acetylation, glycosylation, and phosphorylation processes. Our results indicate that the number of DEGs related with methylation was the largest among the protein modification genes, whereas SUMOylation and ubiquitination did not reveal any significant differences. Although multi-omics studies of fungi can quickly obtain a lot of information, more experimental studies are still needed to evaluate the biological effects of these genes.

After searching for genes with relatively large differential expression in the network, we identified that the functions of most genes have not been individually studied in *C. auris* before. Consequently, we searched their orthologous genes in other *Candida* species and yeasts in Pubmed, CGD (Candida Genome Database), and SGD (Saccharomyces Genome Database). *FCY2* participated in the process of transmembrane transporting and converting 5-fluorocytosine (5FC) into toxic 5-fluorouracil (5FU). Furthermore, FCY2 mutations can mediate resistance to 5FC in both *Candida* species and Cryptococcus (Billmyre et al., 2020). In this study, although we did not know whether genetic mutations are present, we found that FCY2 (B9J08_002435) was upregulated in the resistant *C. auris* strain, a finding that is consistent with the drug sensitivity results obtained for 5FC (MIC≤0.06 μg/ml). *EBP1* was also upregulated in the resistant *C. auris* strain, and previous studies identified that it plays an essential role in the yeast-to-hypha transition (Kurakado et al., 2017). Furthermore, pertaining to genes involved in CO_2_ signaling in fungi, *NCE103* and *UME6* were upregulated in the resistant *C. auris* strain. *NCE103* and *UME6* not only are essential for ensuring the carbon supply required for cell metabolism but also play an important role in signal transduction process such as fungi’s morphology and communication (Martin et al., 2017; Lu et al., 2019). Therefore, we believe that these two genes are perhaps involved in the drug resistance mechanism, yet their specific roles need to be further determined. Among the downregulated genes, we found certain genes or orthologous genes with transmembrane transporter activity, including B9J08_005345, B9J08_004188, B9J08_005571, B9J08_00002660, and B9J08_004099. These transmembrane transporter genes may reduce drug uptake and lead to drug resistance through a different approach than drug efflux. In addition, *ADY2*, which is assigned to the acetate uptake transporter family, was found to be downregulated in the resistant *C. auris* strain. So far, no pleiotropic drug resistance (PDR) transporters belonging to the ABC superfamily exist, which are found to be related with the export of carboxylates under acid stress conditions (Alves et al., 2020). Our research was also in line with a previous study that verified that PKC1 downregulation leads to defects in the formation of biofilms (Heinisch and Rodicio, 2018). Moreover, B9J08_004787 (orthologous gene: YPR013C) and B9J08_004804 (orthologous gene: At4g20930) downregulation may lead to reduced virulence, which is consistent with the weakened virulence of the resistant strain (Mao et al., 2008; Otzen et al., 2014).

Through transcriptome analysis between resistant and susceptible *Candida auris* strains, our study may provide some novel ideas for future studies with respect to understanding the mechanisms of drug resistance in *Candida auris*. The limitation of this study is that only one drug-resistant *C. auris* strain was compared with one susceptible *C. auris* strain. Future research should be performed to confirm the exact function of a certain DEG involved in molecular mechanism of multidrug resistance.

## Data Availability Statement

The datasets presented in this study can be found in online repositories. The names of the repository/repositories and accession number(s) can be found below: https://www.ncbi.nlm.nih.gov/, PRJNA735406.

## Ethics Statement

The Ethics Committee of The First Affiliated Hospital of Nanchang University (approval no. 2016026) approved of the present study. The participants provided written informed consent to participate in this study.

## Author Contributions

XY, DQ, and NL designed the study. WZ, XL, and YL wrote the manuscript. NL reviewed the manuscript. WY, XH, and SJ were responsible for managing the strains and the subsequent data collection. WZ, YL, and WY prepared the figures and tables. All authors contributed to the article and approved the submitted version.

## Conflict of Interest

The authors declare that the research was conducted in the absence of any commercial or financial relationships that could be construed as a potential conflict of interest.

## Footnotes

1 https://www.cdc.gov/fungal/candida-auris/c-auris-antifungal.html

2 http://www.candidagenome.org/download/chromosomal_feature_files/C_auris_B11221/
