# Supplementary material for: A Comparative Transcriptome Between Anti-drug Sensitive and Resistant Candida auris in China
Source: Front Microbiol. 2021 Jul 16;12:708009. doi: 10.3389/fmicb.2021.708009 (PMC8330549; doi:10.3389/fmicb.2021.708009)
Supplement: Supplementary file 1 [file Presentation_1.PPT]

## Slide 1
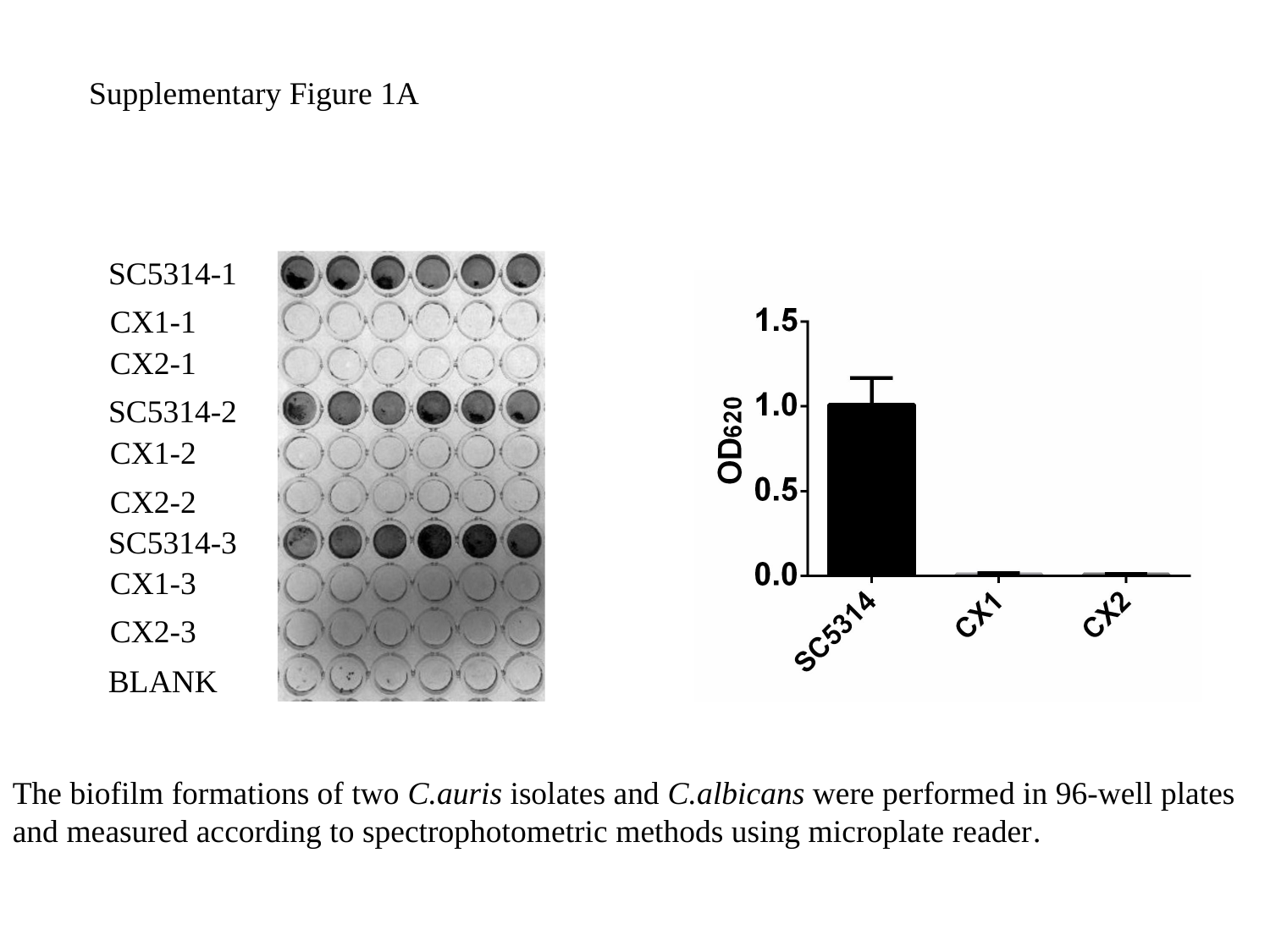

Supplementary Figure 1A
SC5314-1
CX1-1
CX2-1
SC5314-2
CX1-2
CX2-2
SC5314-3
CX1-3
CX2-3
BLANK
The biofilm formations of two C.auris isolates and C.albicans were performed in 96-well plates and measured according to spectrophotometric methods using microplate reader.

## Slide 2
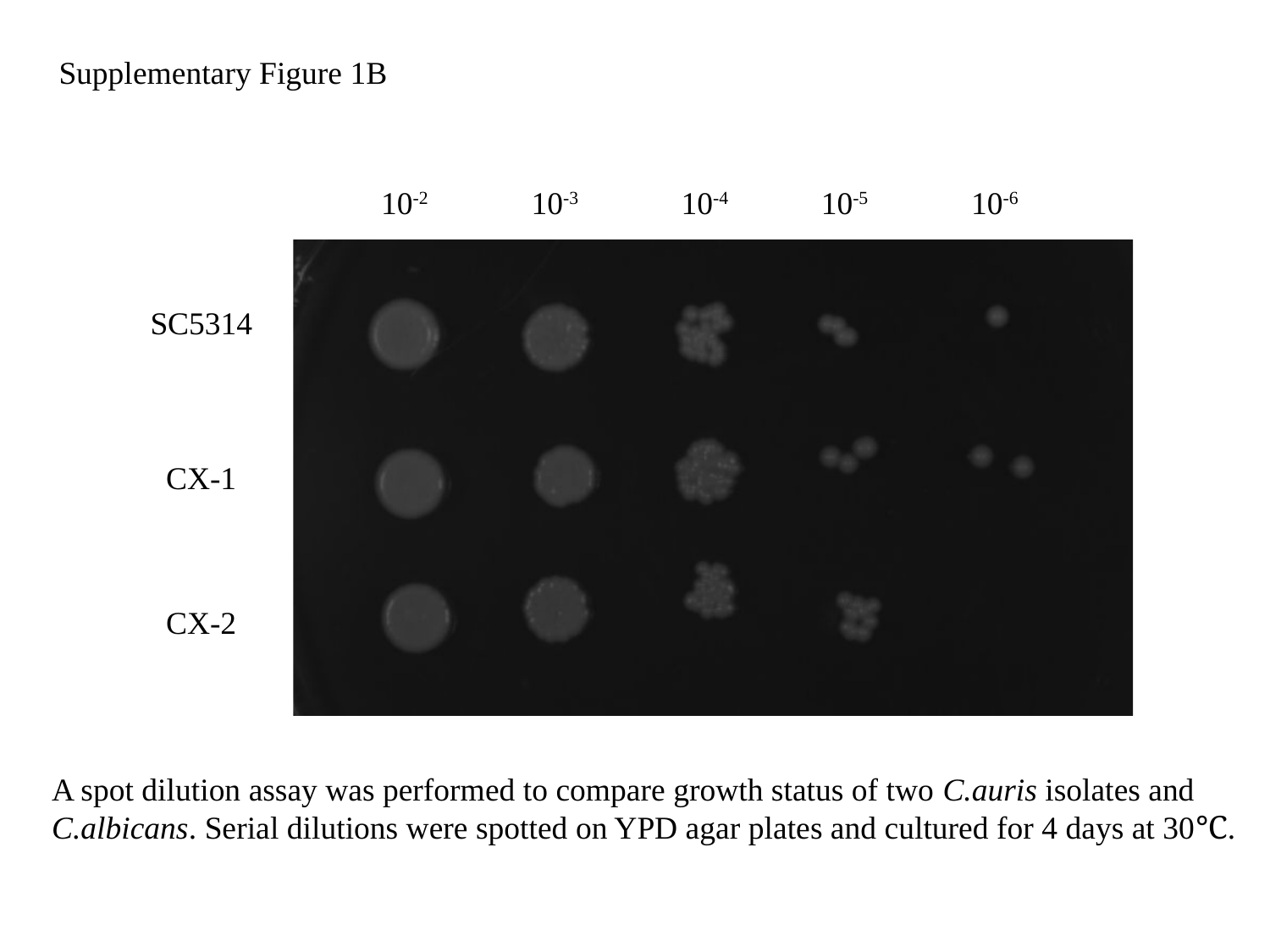

Supplementary Figure 1B
10-2
10-3
10-4
10-5
10-6
SC5314
CX-1
CX-2
A spot dilution assay was performed to compare growth status of two C.auris isolates and C.albicans. Serial dilutions were spotted on YPD agar plates and cultured for 4 days at 30℃.
